# Thermodynamic Mapping of C5‐Substituent Autoxidation Pathways in 1,5‐Dimethylcytosine: A Quantum Chemical Study

**DOI:** 10.1002/jcc.70478

**Published:** 2026-10-01

**Authors:** Vasilii Korotenko, Hendrik Zipse

**Affiliations:** ^1^ Thermal Separation Processes TUHH Hamburg Germany; ^2^ Department of Chemistry LMU München München Germany

## Abstract

The autoxidation pathways of the methyl substituent at position 5 in 1,5‐dimethylcytosine (1,5dmC) were investigated using DFT and DLPNO‐CCSD(T) calculations with implicit SMD(H_2_O) solvation. A radical reaction network was constructed to assess nonenzymatic pathways leading to hydroxymethyl, formyl, and carboxyl oxidation products. The results show that the reactivity of methylated cytosine derivatives is governed not by isolated C—H bond strengths alone, but by the coupled thermodynamics of radical formation, oxygen addition, hydroperoxide formation, and hydroperoxide decomposition. Hydrogen abstraction from the 5‐methyl substituent is the most accessible radical‐forming step in 1,5dmC, whereas protonation increases BDE(C—H) values and suppresses radical formation. Hydroperoxide intermediates play an important role within the considered propagation network, with O—O cleavage being more accessible than O—H cleavage. The calculated reaction free energies rationalize the possible accumulation of the formyl derivative 1m5fC: this formyl derivative is formed through strongly exergonic pathways, whereas its further conversion to the carboxyl derivative 1m5caC is more thermodynamically constrained. Representative transition‐state calculations provide a kinetic consistency check, showing only moderate substrate‐dependent variations in H‐abstraction barriers within each model‐radical class. Overall, the results identify thermodynamically relevant radical intermediates, limiting steps, and product‐forming routes within the considered reaction network for the nonenzymatic autoxidation of methylated cytosine derivatives.

## Graphical abstract

Thermodynamic mapping of autoxidation pathways at the methyl substituent at position 5 in 1,5‐dimethylcytosine reveals a chemically guided radical reaction network connecting the corresponding methyl, hydroxymethyl, formyl, and carboxyl derivatives. Quantum‐chemical calculations identify key radical intermediates, hydroperoxide species, bond dissociation energies, and thermodynamic bottlenecks controlling the nonenzymatic oxidation pathway.

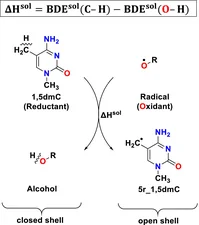

## 1 Introduction

Autoxidation reactions are commonly defined as oxidations that can proceed under the action of molecular oxygen at normal temperatures, without the involvement of a visible flame or an electric spark. Despite this seemingly mild description, the controlled execution of hydrocarbon autoxidation represents a remarkably subtle and challenging problem, one that has attracted growing attention in both academic and industrial research [1, 2]. These reactions form a fundamental class of oxidative processes in which organic compounds interact with molecular oxygen through radical chain mechanisms operating under surprisingly gentle conditions. Notably, autoxidation chemistry plays a central role across a wide range of contexts, from polymer degradation and atmospheric chemistry to biologically relevant oxidation processes [1, 3].

In the present work, we focus on the calculated enthalpies and free energies associated with the autoxidation of 1,5‐dimethylcytosine (1,5dmC). Experimental studies have shown that 5‐methyl‐2′‐deoxycytidine (5mdC) can undergo sequential oxidation by air to form 5‐hydroxymethyl‐2′‐ deoxycytidine (5hmdC), 5‐formyl‐2′‐deoxycytidine (5fdC), and ultimately 5‐carboxy‐2′‐deoxycytidine (5cadC) [4] (Figure 1). In the compound abbreviations used below, m, hm, f, dhm, hp, ca, and pca denote methyl, hydroxymethyl, formyl, dihydroxymethyl (hydrate), hydroperoxy, carboxy, and percarboxylic‐acid C5 substituents, respectively; 1m denotes N1‐methylation. The experimental study reported in reference [4] also provides kinetic data for the stepwise oxidation of 5mdC under aerobic conditions. Monitoring the reaction over time showed that the initial oxidation of 5mdC to 5hmdC is very slow, whereas the subsequent oxidation of 5hmdC to 5fdC is substantially faster. In contrast, further oxidation of 5fdC to 5cadC is inefficient, and only small amounts of the final product are formed under the investigated conditions. At pH 7.4 and 60°C, the reported pseudo‐first‐order rate constant is 4.7 ± 0.6 × 10^−11^ s^−1^ for the 5mdC → 5hmdC transformation, whereas the 5hmdC → 5fdC oxidation proceeds much faster, with *k* = 2.2 ± 0.2 × 10^−8^ s^−1^ at 60°C and *k* = 1.3 ± 0.1 × 10^−8^ s^−1^ after extrapolation to 37°C. The corresponding activation energy for the 5hmdC → 5fdC process was estimated to be 20.8 ± 2.2 kJ mol^−1^. For the 5fdC → 5cadC oxidation, no reliable rate constant was reported because the data deviated substantially from linearity; after 60 h at 60°C, the yield of 5cadC was only 0.03%. These observations show that the oxidation sequence proceeds through kinetically distinct steps and suggest that the formation and reactivity of radical intermediates may play an important role in controlling the overall oxidation pathway.

**FIGURE 1:**
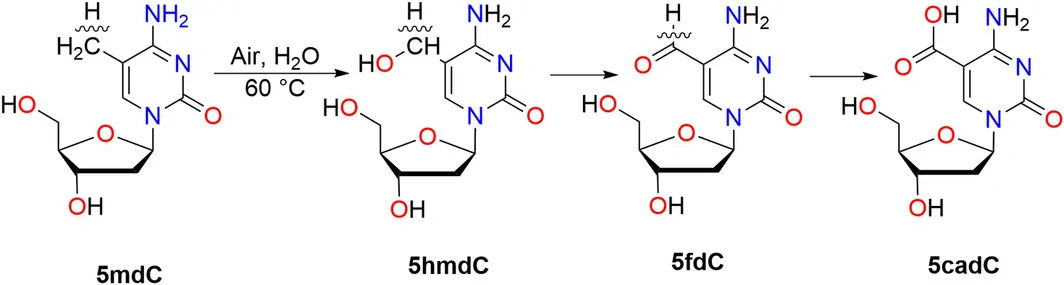
Experimentally observed oxidation products of 5‐methyl‐2′‐deoxycytidine reported in reference [4]. The scheme illustrates the sequential oxidation pathway leading from 5mdC to 5hmdC, 5fdC, and ultimately 5cadC under aerobic conditions.

At first glance, these experimental observations are difficult to rationalize using simple C—H bond‐strength arguments alone. The oxidation of the hydroxymethyl derivative to the formyl derivative proceeds faster than the oxidation of the parent methyl group, although a simple comparison of isolated C—H bond dissociation energies would not directly predict such behavior. Moreover, further oxidation of the formyl derivative is observed even though the formyl C—H bond is expected to be comparatively strong. According to previously reported estimates of C—H bond strengths for related systems [5], the qualitative trend follows CH_2_OH < CH_3_ < CHO, indicating that hydrogen abstraction from the formyl group should be thermodynamically less favorable. The experimentally observed oxidation sequence therefore cannot be explained solely by individual C—H bond dissociation energies. This apparent discrepancy suggests that the thermodynamics of the subsequent radical, peroxyl, and hydroperoxide intermediates may also play an important role in controlling the overall oxidation pathway.

A mechanistic understanding of these transformations is useful for assessing the intrinsic chemical reactivity of methylated cytosine derivatives outside enzymatic environments and for placing such reactivity in the broader context of oxidative DNA methylation chemistry [9] (Figure 2). Within biological chemistry, oxidation reactions of nucleic acid components are most often discussed in the context of enzymatic pathways [10, 11]. A prominent example is the oxidative demethylation of 5‐methylcytosine, which proceeds through a well‐established sequence of hydroxymethyl, formyl, and carboxyl derivatives [12, 13]. While the enzymatic mechanisms responsible for these transformations have been studied in great detail [14, 15], considerably less attention has been paid to the intrinsic chemical reactivity of methylated nucleobases toward nonenzymatic, radical‐mediated oxidation processes [16]. Experimental observations show that methylated cytosine derivatives can undergo slow oxidation in air even in the absence of enzymes or metal‐based oxidants [4, 17]. This raises the question of whether concepts from classical autoxidation chemistry can help rationalize the intrinsic nonenzymatic oxidation behavior of such systems [18].

**FIGURE 2:**
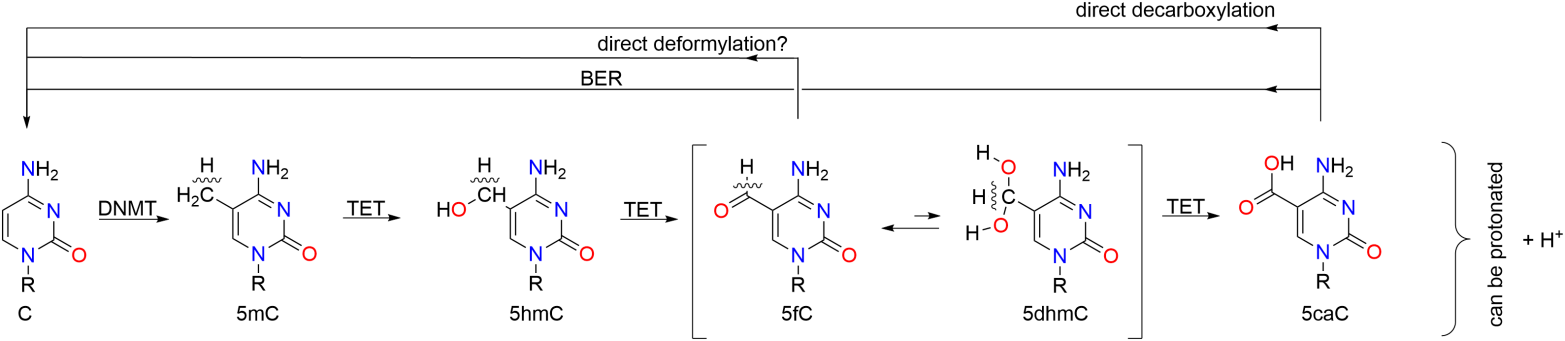
Cytosine derivatives formed during epigenetic gene regulation and their possible further transformations. The wavy line indicates the C—H bonds for which homolytic bond dissociation enthalpies (BDEs) were calculated. BER, base excision repair [6]; DNMT, DNA methyltransferases [7]; TET, ten‐eleven translocation enzymes [8]. R denotes the point of attachment to DNA. The bracketed structures show the equilibrium between 5fC and its hydrated/dihydroxymethyl form 5dhmC. Upper arrows indicate possible removal pathways, including BER, direct deformylation, and direct decarboxylation.

Recent experimental and computational studies have demonstrated that oxidation products of methylated cytosine derivatives, commonly discussed in a biological context, can also be generated in biomimetic systems employing iron‐based oxidants. Such studies highlight that chemically accessible oxidation pathways may lead to products similar to those observed in enzymatic processes, even outside a strictly biological environment [19, 20]. At the same time, the thermodynamic feasibility and mechanistic features of nonenzymatic autoxidation pathways of methylated cytosine derivatives, in particular under metal‐free conditions, remain largely unexplored. Addressing this gap, the present work focuses on the intrinsic thermodynamics of the autoxidation of 1,5‐dimethylcytosine and assesses the viability of radical‐mediated oxidation pathways under aqueous conditions.

Autoxidation reactions are classically described in terms of chain initiation, chain propagation, chain branching, and chain termination steps. In a generic organic substrate R–H, initiation produces a carbon‐centered radical R•. This radical can rapidly add molecular oxygen to form a peroxyl radical ROO•, which can then abstract a hydrogen atom from another substrate molecule to give a hydroperoxide ROOH and regenerate R•. Hydroperoxide decomposition may further produce alkoxyl and hydroxyl radicals, thereby contributing to chain branching. While the propagation, branching, and termination stages are generally considered to be reasonably well understood, the nature of the primary chain initiation reactions remains one of the most persistent open questions in autoxidation chemistry [21]. Moreover, the thermodynamic feasibility of commonly proposed initiation pathways, such as direct reaction with triplet oxygen or unimolecular hydroperoxide decomposition, has repeatedly been called into question [22, 23].

In this context, toluene represents a useful reference system for the present study. Although toluene and 1,5‐dimethylcytosine differ substantially in polarity, heteroatom content, and solvation behavior, both contain a methyl group attached to an aromatic or heteroaromatic π‐system. In toluene, hydrogen abstraction occurs from the benzylic C—H bond to form a benzyl radical, whereas in 1,5‐dimethylcytosine the analogous process involves hydrogen abstraction from the 5‐methyl substituent attached to the heteroaromatic cytosine ring. Thus, the comparison with toluene is not meant to imply identical chemistry, but rather to provide a chemically relevant benchmark for methyl‐group autoxidation adjacent to an aromatic π‐system.

Zipse and Sandhiya investigated several possible initiation reactions for hydrocarbon autoxidation using toluene as a model system. In their study, possible initiation reactions included: (a) unimolecular dissociation of benzyl hydroperoxide into benzyloxyl and hydroxyl radicals, (b) bimolecular hydroperoxide decomposition yielding benzyloxyl and benzylperoxyl radicals together with water, and direct reactions of toluene with either (c) triplet oxygen or (d) ozone [24]. Employing CBS‐QB3, G4, and G3B3 calculations, they demonstrated that initiation by triplet oxygen (c) and unimolecular hydroperoxide decomposition (a) are thermodynamically unfavorable, with strongly positive reaction enthalpies of ΔH_298_(c) = 169.9 ± 2.0 kJ mol^−1^ and ΔH_298_(a) = 202.0 ± 11.6 kJ mol^−1^. In contrast, bimolecular hydroperoxide decomposition (b) was found to be the least endothermic of these pathways, ΔH_298_(b) = 65.8 ± 14.8 kJ mol^−1^, and therefore the most favorable among the three, although it still remains thermodynamically uphill [24]. Historically, autoxidation chains were assumed to be initiated by thermal decomposition of hydroperoxides already present in the substrate [25]. If this picture were fully correct, thermal oxidation of a given organic material would be expected to be highly reproducible. However, this is not observed experimentally, as demonstrated by Gugumus in 1998 [21]. He showed that the lifetime of polypropylene decreases markedly at elevated ozone levels and concluded that hydroperoxide‐initiated chain oxidation primarily governs the oxidation rate only after the induction period. This behavior is believed to extend to other organic materials as well. Taken together with the results of Zipse and Sandhiya, these findings strongly suggest that direct reactions of hydrocarbons with ozone may play a substantial role in chain initiation.

From a mechanistic standpoint, the autoxidation of methylated nucleobases presents a number of fundamental challenges. Their molecular framework differs significantly from that of typical hydrocarbon substrates due to the presence of heteroatoms, polar functional groups, and pronounced solvation effects [20].

Specific microsolvation may substantially affect reaction energy profiles, especially when water directly participates in the mechanism or stabilizes a polar or charged transition state. Such effects have been reported for “on‐water” Diels–Alder reactions, where water stabilizes a polarized TS through hydrogen bonding [26], for aromatic H/D exchange, where a water chain participates in a proton‐transfer/tautomerization pathway [27], and for the reaction of HO• with guanine, where the mechanism involves OH addition, a polar ion‐pair structure G^•+^···OH^−^, and deprotonation [28]. These examples illustrate that explicit water effects are strongest in polar, ion‐pair, or water‐assisted mechanisms.

In contrast, the key steps considered in the present work are neutral radical hydrogen‐atom abstraction reactions, where no localized charge is generated and the main species are neutral closed‐shell molecules and neutral open‐shell radicals. Our previous studies also support this distinction. Hydrogen bonding is a classical polar non‐covalent interaction with a substantial electrostatic component and can therefore affect the relative stabilization of molecular and radical species [29]. In oxygen‐centered radicals, such hydrogen‐bonding effects were shown to alter the relative thermodynamic stability of alcohol/radical pairs [30]. By contrast, one explicit water molecule has a much stronger effect on computed oxidation potentials of cytosine and uracil derivatives because it stabilizes radical cations with localized positive charge [31].

First, the thermodynamics of oxidation depend critically on the stability of the oxidizing species, which in the present case are oxygen‐centered radicals. As established in reference [30], hydrogen bonding can increase the BDE(O—H) values in alcohols, rendering the corresponding RSE(RO•) values less negative and the radicals themselves less stable. These effects are expected to influence both radical stability and hydrogen atom transfer (HAT) energetics, which together govern the thermodynamics of autoxidation.

Second, the thermodynamics of oxidation are governed by the bond dissociation energies of the C—H bonds in the species being oxidized. In the context of methylated cytosine derivatives, variations in substituent type and charge state are expected to significantly affect C—H bond strengths and, consequently, the thermodynamic driving forces of HAT steps. Protonation equilibria under aqueous conditions may further modulate reactivity by altering C—H bond dissociation energies, thereby potentially suppressing or enhancing oxidation processes depending on pH [9, 24].

A representative example of such an elementary step is a HAT reaction between a closed‐shell methylated nucleobase and an oxygen‐centered radical, as schematically illustrated in Figure 3. In this process, a closed‐shell reductant and a radical oxidant are converted into an alcohol and an open‐shell radical derived from 1,5‐dimethylcytosine, highlighting the fundamental change in electronic structure associated with HAT.

**FIGURE 3:**
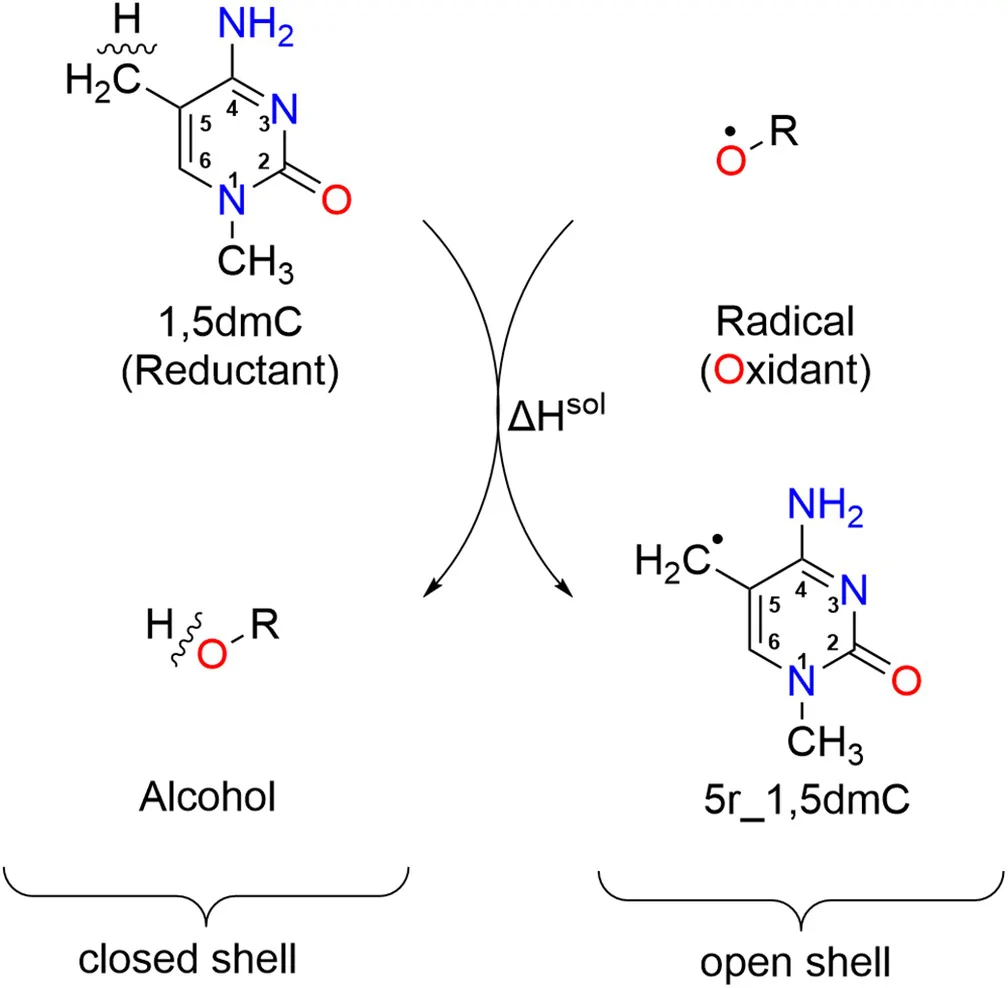
Schematic representation of an elementary hydrogen atom transfer (HAT) step between the closed‐shell reductant 1,5‐dimethylcytosine (1,5dmC) and an oxygen‐centered radical oxidant. The reaction yields the corresponding alcohol and the open‐shell radical 5r_1,5dmC. The thermodynamics of the process in solution are governed by the difference between the C—H and O—H bond dissociation energies, ${\mathrm{\Delta H}}^{\mathrm{sol}}$.

From a thermodynamic perspective, the reaction enthalpy of such a HAT step is determined by the difference between the bond dissociation energies of the bonds being broken and formed. In solution, the corresponding reaction enthalpy can be expressed as follows:

$${\mathrm{\Delta H}}^{\mathrm{sol}}={\mathrm{BDE}}^{\mathrm{sol}}\left(C–H\right)-{\mathrm{BDE}}^{\mathrm{sol}}\left(O–H\right)$$

where ${\mathrm{BDE}}^{\mathrm{sol}}\left(C–H\right)$ refers to the solution‐phase bond dissociation energy of the abstracted C—H bond in the methylated nucleobase, and ${\mathrm{BDE}}^{\mathrm{sol}}\left(O–H\right)$ corresponds to the solution‐phase bond dissociation energy of the newly formed O—H bond in the alcohol product. This relation emphasizes that the thermodynamic feasibility of HAT steps in autoxidation is governed by a balance between radical stability and bond strengths, both of which may be affected by solvation.

To address these challenges, 1,5‐dimethylcytosine was selected as a minimal model system that retains the essential chemical features of methylated cytosine while avoiding additional complications associated with the sugar moiety or the DNA backbone. This model enables a systematic analysis of radical formation and selected propagation steps within a well‐defined framework and allows for direct comparison with classical hydrocarbon autoxidation mechanisms.

In the present work, the thermodynamics of the autoxidation of 1,5‐dimethylcytosine are investigated using density functional theory and high‐level DLPNO‐CCSD(T) calculations with implicit solvation. Particular emphasis is placed on evaluating the feasibility of selected initiation pathways [24], identifying thermodynamically limiting steps within radical chain cycles, and assessing the role of HAT and bond dissociation energies in governing reactivity. By comparing the resulting reaction energetics with available experimental oxidation trends [4], this study aims to assess whether selected nonenzymatic autoxidation pathways of methylated cytosine derivatives are thermodynamically plausible and to identify mechanistic features that may distinguish these processes from classical hydrocarbon systems.

The aim of this work is not to construct a complete kinetic model of all possible radical reactions in solution. Instead, we focus on a chemically guided thermodynamic reaction network that connects 1,5‐dimethylcytosine with experimentally observed oxidation products of 5‐methylcytosine derivatives. The considered reaction network should therefore be understood as a thermodynamic feasibility analysis of selected initiation and propagation reactions, rather than as a mechanistic description or an exhaustive enumeration of all possible radical processes.

## 2 Computational Details

In this work, all geometries were optimized at the SMD(H_2_O)/(U)B3LYP‐D3/6–31+G(d,p) level of theory with the SMD implicit solvent model [32] using Gaussian 09, Revision D.01 [33]. Grimme's D3 dispersion correction was included in all geometry optimizations to account for dispersion interactions [34, 35].

Since the relative tautomeric stability of the studied molecules is not trivial and can be affected by solvation effects, all chemically reasonable tautomeric forms were considered explicitly. Initial tautomeric structures were generated manually by systematic relocation of labile acidic hydrogen atoms to the available heteroatom lone‐pair sites of each cytosine derivative. The substitution pattern of each molecule was taken into account; for example, proton transfer to a nitrogen (N1) atom already substituted by a methyl group was not considered. No automated tautomer‐generation software was used. Using the binomial coefficient, $C_{n}^{k}=\frac{n!}{\left(n-k\right)!\cdot k!}$, one can estimate the number of possible tautomers of an asymmetric molecule having (*n*) available lone‐pair acceptor sites and (*k*) labile acidic protons (*k* ≤ *n*). No preliminary selection based on canonical structures or expected stability was applied. Instead, all generated chemically meaningful tautomers were optimized. The individuality of the found tautomers and their conformers was confirmed using an energy criterion (∆E_tot_ > 10^−7^ a.u.) and by comparing geometries through distances between each atom and the centroid point [30].

Frequency calculations were carried out to verify that the optimized structures are true minima without imaginary vibrational frequencies. Thermochemical corrections to $H^{\mathrm{sol}}$ and $G^{\mathrm{sol}}$ at 298.15 K were calculated with GoodVibes using the quasi‐harmonic approximation [36]. A “solution” symbol (^sol^) denotes a standard state of 1 mol L^−1^ in the solution‐phase, whereas a “gas” symbol (^gas^) denotes a standard state of 1 atm in the gas‐phase. For each studied nucleobase, all stable tautomeric and conformational minima were included in the calculation of Boltzmann‐weighted $H^{\mathrm{sol}}$ and $G^{\mathrm{sol}}$ values.

The single‐point energies were also calculated for the optimized geometries at the DLPNO‐CCSD(T) level [37, 38, 39]. Two‐point (cc‐pVTZ and cc‐pVQZ) extrapolation was employed at the DLPNO‐CCSD(T) level of theory to estimate a result obtained using a complete (infinitely large) basis set [38, 40]. Standard state solvation free energies ${\mathrm{\Delta G}}^{\text{solv}}$ were computed as the difference between the solution‐phase free energy $G^{\mathrm{sol}}$ of the solution‐phase optimized molecule (SMD(H_2_O)/(U)B3LYP‐D3/6–31+G(d,p)), and the gas‐phase single‐point free energy $G^{\mathrm{gas}}$ ((U)B3LYP‐D3/6–31+G(d,p)//SMD(H_2_O)/(U)B3LYP‐D3/6–31+G(d,p))

$${\mathrm{\Delta G}}^{\text{solv}}=G^{\mathrm{sol}}-G^{\mathrm{gas}}$$

The solution‐phase single‐point free energies $G^{\mathrm{sol}}$ are computed by combining the gas‐phase free energies $G^{\mathrm{gas}}$ with the solvation free energies ${\mathrm{\Delta G}}^{\text{solv}}$

$$\begin{array}{c} G^{\mathrm{sol}}=E_{\mathrm{tot}}^{\text{single}-\text{point}}+{\mathrm{ZPE}}^{\mathrm{SMD}/\left(U\right)B3\mathrm{LYP}-D3}+{\mathrm{\delta G}}_{0K\to 298K}^{\mathrm{SMD}/\left(U\right)B3\mathrm{LYP}-D3}\\ \;{+\mathrm{\Delta G}}_{298K}^{1\mathrm{atm}\to 1M}+{\mathrm{\Delta G}}^{\text{solv}} \end{array}$$

${\mathrm{\Delta G}}_{298K}^{1\mathrm{atm}\to 1M}$ = 7.91 kJ mol^−1^ corresponds to $\mathrm{RT}\cdot \ln\left(24.46\right)$ at 298.15 K and accounts for converting the gas‐phase standard state (1 atm) to the solution standard state (1 mol L^−1^), since the concentration of an ideal gas at 1 atm and 298.15 K is 0.0409 mol L^−1^.

Initial structures for the H‐abstraction transition‐state searches were prepared by a conformational search of pre‐reactive substrate–radical complexes in Maestro/MacroModel using the OPLS4 force field and water as solvent. Mixed torsional/low‐mode sampling was used with the multi‐ligand option, extended torsional sampling, 100 steps per rotatable bond, a maximum of 1000 search steps, and an energy window of 50 kJ mol^−1^ for retaining structures. During the conformational search, distance and angular restraints were applied to keep the attacking radical in a chemically meaningful orientation with respect to the abstracted hydrogen atom: The O•···H distance between the attacking oxygen atom and the abstracted hydrogen was restrained to approximately 2.0 Å with a force constant of 100, and a C—O•···H angle of approximately 109° was imposed to maintain a suitable approach geometry.

The resulting complexes were inspected and refined iteratively. For each pre‐reactive complex, the hydrogen atom to be abstracted was identified as the C‐bound hydrogen closest to the selected attacking oxygen atom. The corresponding C—H bond was elongated to 1.20 Å, while the forming O—H distance was adjusted toward 1.40 Å. During this refinement, the attacking radical was moved as a rigid fragment relative to the substrate, while preserving its internal geometry. All lateral, nonreactive intermolecular contacts were checked at each step, and only the selected forming O—H contact was allowed to be shorter than the minimum intermolecular cutoff. Geometries with nonreactive intermolecular contacts shorter than 1.70 Å were iteratively corrected by moving the attacking fragment away from the substrate and, when necessary, by testing alternative O—H approach directions. The final structures therefore satisfied the target reactive C—H/O—H distances while avoiding artificial steric clashes between the substrate and the attacking radical, and were used as starting points for subsequent TS optimizations. All optimized transition‐state structures were verified by frequency calculations and showed a single imaginary frequency corresponding to the intended hydrogen‐transfer coordinate.

## 3 Computational Results

### 3.1 BDE(C—H)

To justify the focus on hydrogen abstraction from the 5‐methyl group, preliminary absolute solution‐phase BDE calculations, ${\mathrm{BDE}}_{\mathrm{abs}}^{\mathrm{sol}}$ (RH → R• + H•), were performed for alternative hydrogen‐abstraction sites in 1,5‐dimethylcytosine (Figure 4) at the SMD/DFT level. In contrast to the calibrated ${\mathrm{BDE}}_{\mathrm{rel}}^{\mathrm{sol}}$ values discussed below, these absolute ${\mathrm{BDE}}_{\mathrm{abs}}^{\mathrm{sol}}$ values were calculated directly from the homolytic bond cleavage reaction. The calculated values are 404.8 kJ mol^−1^ for position 1, 439.4 kJ mol^−1^ for the N–H bond at position 4, 487.0 kJ mol^−1^ for the C—H bond at position 6, and 381.0 kJ mol^−1^ for the C–H bond of the 5‐methyl group. Thus, the 5‐methyl C—H bond is the weakest among the considered sites. Its BDE is lower by 23.8, 58.4, and 106.0 kJ mol^−1^ compared with positions 1, 4, and 6, respectively. These results support hydrogen abstraction from the 5‐methyl group as the most thermodynamically accessible radical‐forming step among the sites considered and justify the mechanistic focus adopted in this work. The prefixes p_, 5r_, 5hor_, and 5hoor_ denote protonated species, carbon‐centered radicals, alkoxyl radicals, and peroxyl radicals, respectively.

**FIGURE 4:**
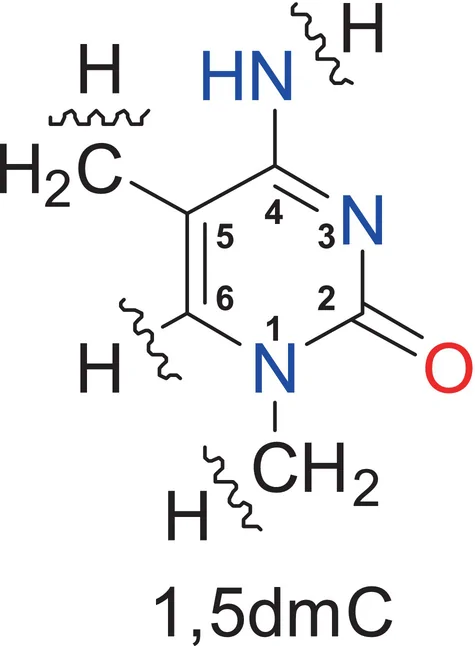
Possible hydrogen‐abstraction sites considered for 1,5‐dimethylcytosine (1,5dmC). Absolute ${\mathrm{BDE}}_{\mathrm{abs}}^{\mathrm{sol}}$(R–H) values were calculated for positions 1, 4, 5, and 6 to compare the thermodynamic accessibility of radical formation at different sites.

Calculated relative solution‐phase C–H bond dissociation energies, ${\mathrm{BDE}}_{\mathrm{rel}}^{\mathrm{sol}}$(C—H), for the investigated cytosine derivatives are summarized in Table 1. These values correspond to homolytic cleavage of the C—H bond in the substituent at position 5 and were obtained at the SMD/CC level. The ${\mathrm{BDE}}_{\mathrm{rel}}^{\mathrm{sol}}$ values were derived from radical stabilization energies using the experimental benzylic C—H bond dissociation energy of toluene as the reference value [41]. Thus, for a given carbon‐centered radical R•, the corresponding C—H bond strength was estimated as: ${\mathrm{BDE}}_{\mathrm{rel}}^{\mathrm{sol}}\left(R–H\right)=\mathrm{RSE}\left(R•\right)+{\mathrm{BDE}}_{\exp}\left({\text{PhCH}}_{2}–H\right).$

**TABLE 1:** Radical stabilization enthalpies, RSE, and radical stabilization free energies, RSFE, calculated for the investigated bond‐dissociation processes.

Molecule	Bond(X—H)	SMD/DFT			SMD/CC			${\mathrm{BDE}}_{\exp}$
		RSE	RSFE	${\mathrm{BDE}}_{\mathrm{rel}}^{\mathrm{sol}}$	RSE	RSFE	${\mathrm{BDE}}_{\mathrm{rel}}^{\mathrm{sol}}$	
1,5dmC	C—H	5.6	4.4	380.6	12.6	11.4	387.6	375 (PhCH_2_—H) [41]
1m5hmC	C—H	−19.0	−23.9	356.0	−6.4	−9.5	368.6	
1m5fC	C—H	23.3	19.5	398.3	25.4	21.6	400.4	
1m5dhmC	C—H	−69.7	−76.4	305.3	−51.8	−57.2	323.2	
p_1,5dmC	C—H	13.8	13.1	388.8	26.0	24.2	401.0	
p_1m5hmC	C—H	−18.0	−21.1	357.0	−3.2	−6.0	371.8	
p_1m5fC	C—H	29.9	26.3	404.9	28.3	24.5	403.3	
p_1m5dhmC	C—H	−15.7	−20.7	359.3	−1.6	−6.5	373.4	
1m5hpmC	C—H	2.7	−1.9	377.7	10.3	5.7	385.3	
1m5hphmC	C—H	18.6	16.6	393.6	32.5	30.2	407.5	
1m5hmC	O—H	−65.6	−71.6	431.4	−51.6	−54.7	445.4	497 (HO—H) [42]
1m5dhmC	O—H	−69.1	−72.6	427.9	−46.9	−48.7	450.1	
1m5caC	O—H	−59.2	−65.2	437.8	−8.4	−14.0	488.6	
1m5hphmC	O—H	−64.8	−69.1	432.2	−34.1	−37.9	462.9	
1m5hpdhmC	O—H	−76.0	−79.4	421.0	−36.1	−41.1	460.9	
1m5hpmC	OO—H	−21.1	−22.2	475.9	−13.2	−14.1	483.8	
1m5hphmC	OO—H	−16.4	−15.4	480.6	−6.2	−5.8	490.8	
1m5hpdhmC	OO—H	−12.5	−13.7	484.5	−3.0	−2.2	494.0	
1m5pcaC	OO—H	21.0	21.5	518.0	33.5	33.8	530.5	
1m5hpmC	O—O	−31.7	−43.1	181.3	−19.1	−28.6	193.9	213 (HO—OH) [43]
1m5hphmC	O—O	−41.2	−48.4	171.8	−19.8	−27.3	193.2	
1m5hpdhmC	O—O	−53.3	−62.3	159.7	−18.7	−26.1	194.3	
1m5pcaC	O—O	−64.9	−73.6	148.1	−10.8	−20.1	202.2	

*Note:* RSE values refer to the 1 atm standard state, whereas RSFE values refer to the 1 mol L^−1^ standard state. Calculations were performed at the SMD/DFT (SMD(H_2_O)/(U)B3LYP‐D3/6–31+G(d,p)) and SMD/CC (SMD(H_2_O)/DLPNO‐CCSD(T)/CBS//SMD(H_2_O)/(U)B3LYP‐D3/6–31+G(d,p)) levels. The considered bond types include C—H, O—H, O—H in hydroperoxides, and O—O bonds. Relative solution‐phase bond dissociation energies, ${\mathrm{BDE}}_{\mathrm{rel}}^{\mathrm{sol}}$, were derived from the calculated radical stabilization energies using experimental gas‐phase reference ${\mathrm{BDE}}_{\exp}$ values at 298 K for appropriate reference molecules. The table also indicates the reference molecule used for each calibration: PhCH_3_ for C—H bonds, H_2_O for O—H bonds, and H_2_O_2_ for O—O bonds. For 1m5hpmC and 1m5hphmC, the reported C−H values are effective energies based on transition‐state structures because no stable carbon‐centered radical minima could be located.

Here, RSE(R•) is the radical stabilization energy relative to the benzyl radical and corresponds to the calculated reaction enthalpy for the reaction R—H + PhCH_2_• → R• + PhCH_3_ and ${\mathrm{BDE}}_{\exp}$ (PhCH_2_—H) is the experimental C—H bond dissociation energy of toluene ${\mathrm{BDE}}_{\exp}$(PhCH_2_—H) = 375 kJ mol^−1^ [41]. This calibration allows the relative radical stabilization energies to be converted into C–H bond strengths that can be compared across the investigated substituents.

A clear dependence of ${\mathrm{BDE}}_{\mathrm{rel}}^{\mathrm{sol}}$(C—H) on the nature and electronic structure of the substituent is observed. The lowest value is obtained for 1m5dhmC, while 1m5hmC shows a higher ${\mathrm{BDE}}_{\mathrm{rel}}^{\mathrm{sol}}$(C—H). The bond becomes stronger for 1,5dmC, and the highest value is observed for 1m5fC. Thus, the following qualitative trend is obtained: 1m5dhmC < 1m5hmC < PhCH_3_ <1,5dmC< 1m5fC (Figure 5). This behavior reflects differences in substituent effects and radical stabilization. For comparison, the calculated C—H bond strength for 1,5dmC is close to the experimental value for the benzylic C—H bond in toluene, indicating comparable thermodynamic stability of the corresponding carbon‐centered radicals.

**FIGURE 5:**
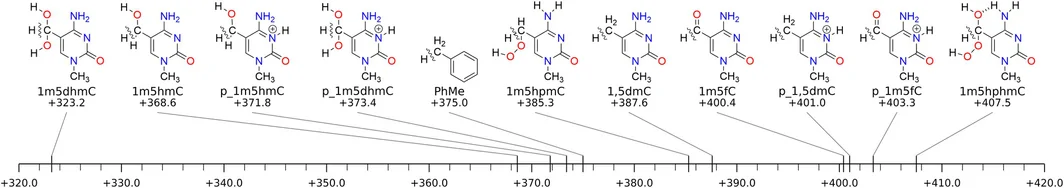
Boltzmann‐averaged BDE(C—H) values calculated at the SMD/CC level. Values are given in kJ mol^−1^.

The particularly low ${\mathrm{BDE}}_{\mathrm{rel}}^{\mathrm{sol}}$(C—H) value obtained for 1m5dhmC deserves special attention. Although 1m5dhmC is formally the hydrated, dihydroxymethyl form of the formyl derivative 1m5fC, hydrogen abstraction from this species leads to a radical that can be strongly stabilized by tautomeric rearrangement. In the present computational protocol, all relevant tautomers and conformers of each closed‐shell molecule and radical were considered, and the reported values correspond to Boltzmann‐averaged thermodynamic quantities. Therefore, the calculated ${\mathrm{BDE}}_{\mathrm{rel}}^{\mathrm{sol}}$(C—H) values include not only the energy required for the formal C—H bond cleavage, but also the subsequent stabilization of the radical by tautomerization.

This effect is illustrated schematically in Figure 6. After initial hydrogen abstraction from the dihydroxymethyl group of 1m5dhmC, the resulting radical can undergo proton transfer from a hydroxyl group to the N3 atom of the cytosine ring. The resulting tautomer is substantially more stable and is characterized by redistribution of spin density over the heteroaromatic framework. One representative resonance structure is shown in Figure 6 to illustrate the dominant spin‐density distribution obtained at the DFT level. At the SMD/DFT level, the RSE value for the final optimized tautomer of the 1m5dhmC‐derived radical is −69.7 kJ mol^−1^, whereas the corresponding value for simple hydrogen abstraction without tautomeric stabilization is only −6.6 kJ mol^−1^. The difference of −63.1 kJ mol^−1^ therefore reflects the additional tautomeric stabilization of this radical. This stabilization explains why the calculated C–H bond strength of 1m5dhmC is much lower than that of the corresponding formyl derivative 1m5fC.

**FIGURE 6:**
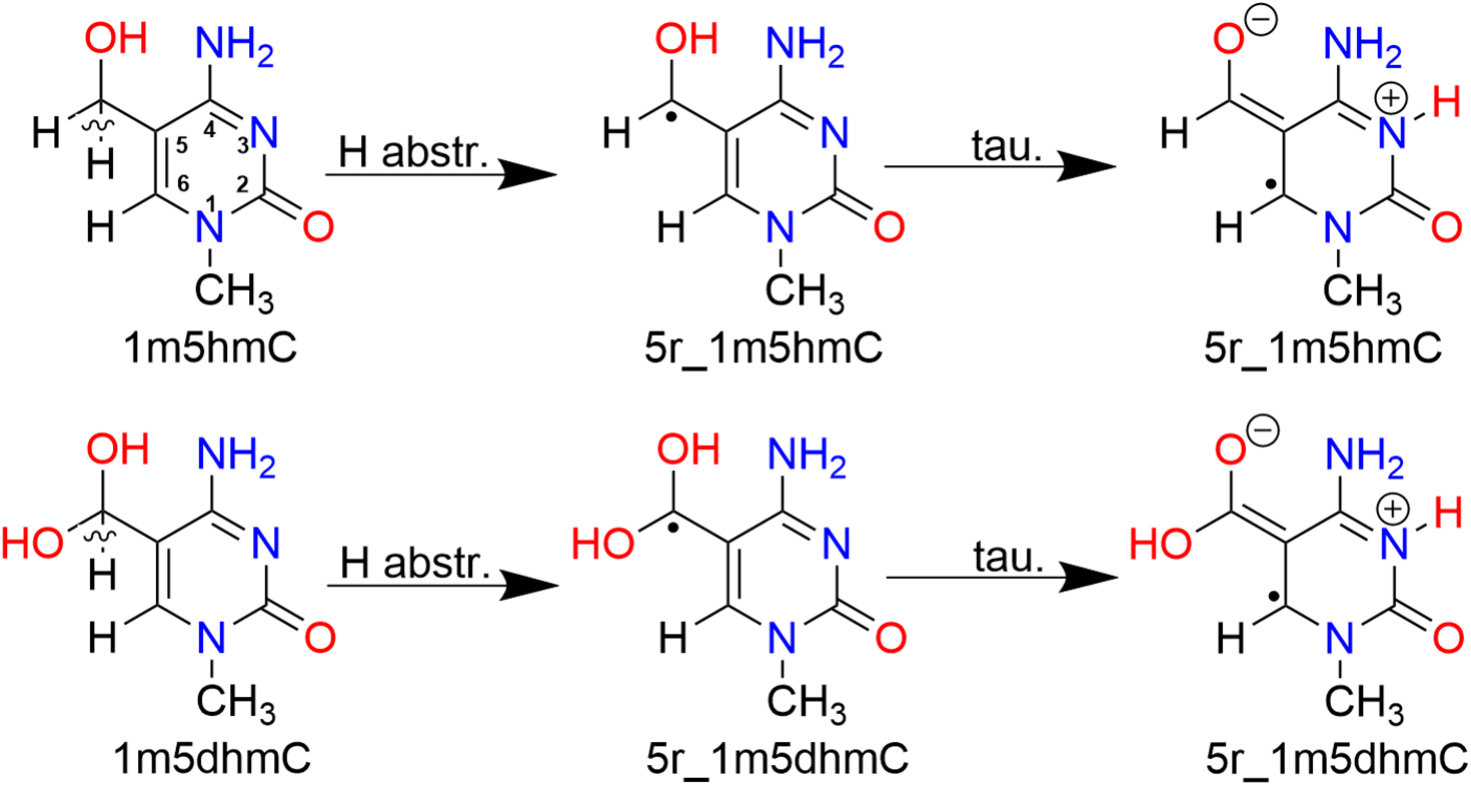
Schematic illustration of radical stabilization after hydrogen abstraction from 1m5hmC (top) and 1m5dhmC (bottom). Initial C–H bond cleavage gives a carbon‐centered radical, which can undergo tautomeric rearrangement by proton transfer from the hydroxyl group to the N3 atom. The resulting tautomer is stabilized by redistribution of spin density over the heteroaromatic framework. The radical dot and formal charges shown in the representative resonance structure schematically reflect the dominant spin‐density localization and NBO charge distribution obtained at the SMD/DFT level. The structure is therefore intended as a schematic representation of the calculated electronic structure rather than as a unique Lewis formula.

A similar, although less pronounced, stabilization effect is also observed for the 1m5hmC‐derived radical. At the SMD/DFT level, the RSE value for simple hydrogen abstraction without tautomeric stabilization is −15.6 kJ mol^−1^, whereas the corresponding value for the final optimized tautomer is −19.0 kJ mol^−1^. The resulting tautomeric stabilization amounts to −3.4 kJ mol^−1^. Thus, tautomeric rearrangement also contributes to the stabilization of the 1m5hmC‐derived radical, but the effect is considerably smaller than for the 1m5dhmC‐derived radical.

Some radical intermediates may undergo tautomeric rearrangement and spin‐density redistribution, which could make alternative oxygen‐addition sites energetically accessible. In the present work, however, we focus on chemically guided side‐chain oxidation pathways leading to the experimentally relevant hydroxymethyl, formyl, and carboxyl products. A complete exploration of ring‐centered oxygen‐addition pathways is beyond the scope of this study.

Nevertheless, once a carbon‐centered radical is generated within the considered side‐chain oxidation network, it can react with triplet molecular oxygen, •OO• (^3^O_2_). Here, •OO• is used as a schematic notation to emphasize the diradical character of triplet oxygen [44]. Addition of •OO• to the radical center leads to the formation of a peroxyl radical intermediate, ROO•, which is a well‐established step in radical oxidation processes. Subsequent hydrogen‐transfer reactions of such peroxyl radicals typically give rise to hydroperoxide species.

To account for this possibility, two hydroperoxide derivatives were examined: 1m5hpmC and 1m5hphmC. These molecules retain a C—H bond in the substituent, allowing the corresponding bond dissociation process to be evaluated for hydroperoxide intermediates. For 1m5hpmC, attempts to optimize the radical produced after homolytic cleavage of the C—H bond did not lead to a stable local minimum. During geometry optimization, the system spontaneously evolved toward fragmentation into HO• and the oxidized nucleobase 1m5fC. This behavior indicates that the corresponding carbon‐centered radical is intrinsically unstable with respect to dissociation of the hydroperoxide fragment. Because of this instability, the bond dissociation process for 1m5hpmC was described using a transition‐state representation corresponding to the radical dissociation pathway, R—H → [R•]^‡^ + H•, rather than a conventional radical minimum, R—H → R• + H•. The transition‐state structure exhibits one imaginary frequency corresponding to O─O bond elongation and subsequent fragmentation toward HO• and 1m5fC; therefore, the reported C─H bond‐dissociation value should be regarded as an effective energy based on this transition‐state structure, rather than as a conventional equilibrium BDE derived from a radical minimum.

A similar situation is observed for the related hydroperoxide 1m5hphmC. In this case, optimization of the radical obtained after C—H bond cleavage also does not converge to a stable minimum. Instead, the structure tends to dissociate into HO• and the oxidized product 1m5caC. As in the previous case, the unstable radical configuration was represented by a transition‐state structure associated with this dissociation process.

—Despite this instability of the isolated radical structures, the effective C−H bond‐dissociation values for the two hydroperoxide derivatives can still be compared within this approximation. The corresponding qualitative relation is 1m5hpmC < 1m5hphmC, which reflects a somewhat stronger C—H bond in the hydroperoxy‐hydroxymethyl derivative.

### 3.2 BDE(O–H)

Calculated ${\mathrm{BDE}}_{\mathrm{rel}}^{\mathrm{sol}}$ (O—H) values for oxygen‐containing derivatives of cytosine are summarized in Table 1. All values correspond to homolytic cleavage of the O—H bond and were obtained at the SMD/CC level.

For hydroxyl‐containing derivatives, the calculated values refer to cleavage of the O—H bond in the hydroxy group. The weakest O—H bond is found for 1m5hmC, while 1m5dhmC shows a higher value. Further oxidation of the substituent leads to stronger bonds in 1m5hpdhmC and 1m5hphmC, whereas 1m5caC exhibits the strongest O—H bond among the cytosine derivatives considered. When compared with water, which has an experimental O—H bond dissociation energy of 497 kJ mol^−1^ [42], the following qualitative trend is obtained: 1m5hmC < 1m5dhmC < 1m5hpdhmC < 1m5hphmC < 1m5caC < H_2_O (Figure 7).

**FIGURE 7:**
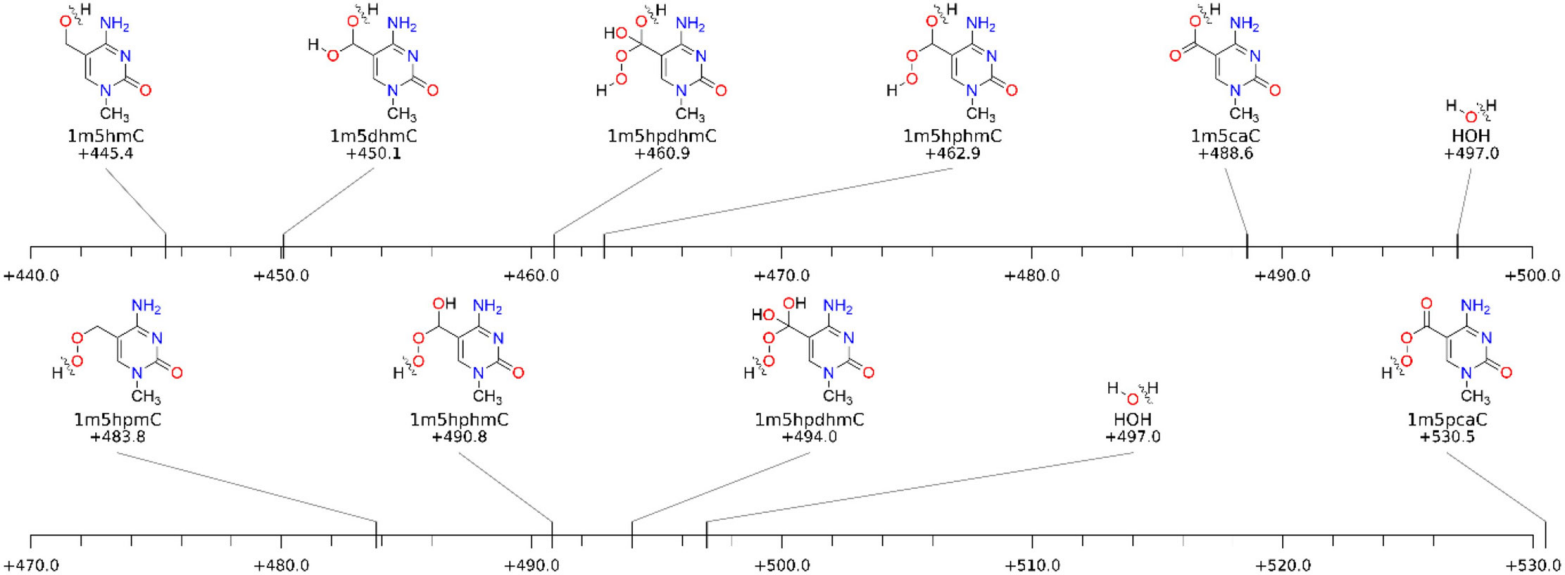
Boltzmann‐averaged BDE(O—H) values calculated at the SMD/CC level. Values are given in kJ mol^−1^.

A separate analysis was carried out for hydroperoxide derivatives containing the OOH fragment. In this case, the calculated values refer to cleavage of the terminal O—H bond in the OOH group. The weakest O—H bond is found for 1m5hpmC, while 1m5hphmC and 1m5hpdhmC show progressively stronger bonds. The O—H bond strength of water remains higher than that of these hydroperoxide derivatives, whereas the percarboxylic acid 1m5pcaC exhibits the strongest O—H bond among the hydroperoxide‐containing species. The corresponding qualitative relation is therefore: 1m5hpmC < 1m5hphmC < 1m5hpdhmC < H_2_O < 1m5pcaC.

### 3.3 BDE(O–O)

Calculated ${\mathrm{BDE}}_{\mathrm{rel}}^{\mathrm{sol}}$(O—O) values for the hydroperoxide intermediates are summarized in Table 1. All values correspond to homolytic cleavage of the O—O bond in hydroperoxy‐containing derivatives and were obtained at the SMD/CC level.

—The O−O bond strength varies only slightly among the studied hydroperoxide species. The O−O bond strengths of 1m5hphmC, 1m5hpmC, and 1m5hpdhmC are very similar, with calculated values of 193.2, 193.9, and 194.3 kJ mol−1, respectively. The strongest O−O bond among the considered intermediates is observed for 1m5pcaC. The resulting qualitative trend is therefore: 1m5hphmC ≈ 1m5hpmC ≈ 1m5hpdhmC < 1m5pcaC < H_2_O_2_ (Figure 8).

**FIGURE 8:**
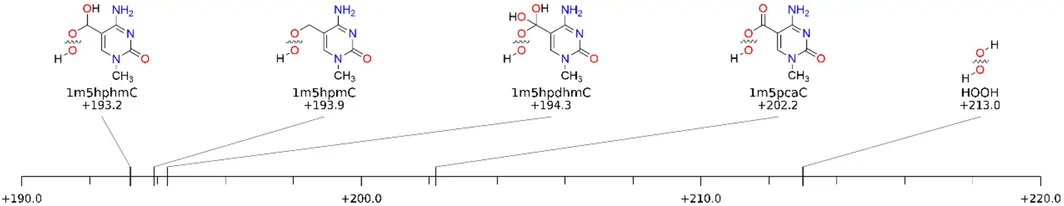
Boltzmann‐averaged BDE(O—O) values calculated at the SMD/CC level. Values are given in kJ mol^−1^.

The calculated values are consistent with the experimental estimate for hydrogen peroxide, for which the O—O bond dissociation energy is 213 kJ mol^−1^ [43].

In comparison with the corresponding O—H bonds discussed above, the O—O bond in the hydroperoxy group is significantly weaker in all considered intermediates. As a result, homolytic cleavage of the O—O bond represents the most thermodynamically accessible bond‐cleavage process in these species and may play an important role in the decomposition pathways of hydroperoxide intermediates during autoxidation.

### 3.4 Initiation and Early Propagation: Formation of 1m5hmC

The elementary steps included in the considered reaction network were selected according to three criteria: (i) They correspond to standard elementary transformations known from radical autoxidation chemistry, such as hydrogen abstraction, oxygen addition, peroxyl‐radical formation, hydroperoxide formation, and hydroperoxide decomposition; (ii) They connect the starting methylated cytosine derivative to experimentally observed oxidation products; and (iii) They involve intermediates whose thermodynamic stability can be evaluated consistently using the same computational protocol.

Possible initiation reactions of the autoxidation process are shown in Figure 9. The corresponding reaction enthalpies and Gibbs free energies for the elementary reactions and cycles discussed in the following sections are summarized in Table 2. For hydrocarbon systems, several types of initiation steps are usually considered: hydrogen atom abstraction by oxygen‐containing radicals, reactions with ozone, and decomposition of hydroperoxides. These processes lead to the formation of a primary radical, which then enters the chain reactions of oxidation [24].

**FIGURE 9:**
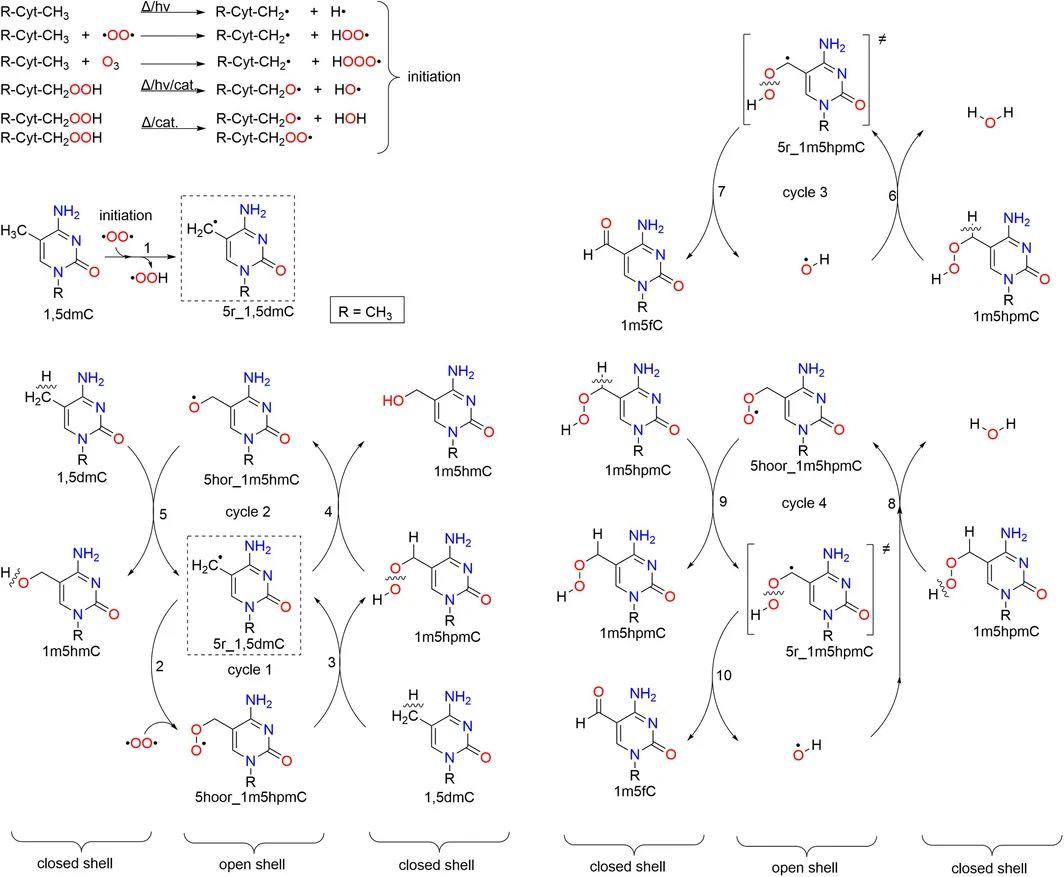
Chemically guided radical reaction network considered for the autoxidation of the substituent at position 5 in 1,5‐dimethylcytosine: Initiation and cycles 1–4. Stable closed‐shell products: 1m5hmC and 1m5fC.

**TABLE 2:** Reaction enthalpies, $\Delta H^{\mathrm{sol}}$, and Gibbs free energies, $\Delta G^{\mathrm{sol}}$, calculated for the studied elementary reactions and reaction cycles.

Cycle	Reaction	Closed‐shell		SMD/DFT		SMD/CC	
		Reactant	Product	$\Delta H^{\mathrm{sol}}$	$\Delta G^{\mathrm{sol}}$	$\Delta H^{\mathrm{sol}}$	$\Delta G^{\mathrm{sol}}$
	1	1,5dmC	—	153.4	146.7	170.6	163.8
1	2	—	—	−105.2	−65.5	−111.4	−71.7
	3	1,5dmC	1m5hpmC	21.7	23.3	9.3	10.8
2	4	1m5hpmC	1m5hmC	−170.8	−173.2	−172.6	−175.3
	5	1,5dmC	1m5hmC	−54.6	−51.5	−67.3	−67.1
3	6	1m5hpmC	HOH	−123.2	−129.4	−121.2	−127.4
	7	—	1m5fC	−158.1	−189.8	−166.4	−198.1
4	8	1m5hpmC	HOH	−141.9	−146.4	−128.2	−132.6
	9	1m5hpmC	1m5hpmC	18.7	17.0	6.9	5.1
	10	—	1m5fC	−158.1	−189.8	−166.4	−198.1
5	11	1m5hmC	1m5hphmC	−7.7	−11.8	−16.7	−18.4
	12	—	—	−104.3	−60.5	−118.1	−74.9
	13	1m5hphmC	1m5fC + HOOH	6.1	−36.1	17.4	−25.1
6	14	1m5hmC + HOOH	1m5dhmC (1m5fC + HOH)	−148.1	−135.8	−171.8	−161.4
	15	1m5hmC	HOH	−144.8	−151.4	−138.0	−142.7
7	16	1m5fC	1m5pcaC	−2.7	−5.2	−24.6	−27.0
	17	—	—	−131.3	−90.3	−138.7	−97.3
8	18	1m5dhmC (1m5fC + HOH)	1m5hpdhmC	−62.3	−66.0	−65.3	−69.6
	19	—	—	−56.2	−9.4	−77.5	−30.3
	20	1m5hpdhmC	1m5caC + HOOH	−42.8	−87.8	−33.2	−76.9
	21	1m5hpdhmC	1m5pcaC + HOH	−37.5	−80.3	−30.9	−71.4
	22	1m5fC + 1m5hpmC	1m5fC_1m5hpmC	−26.1	33.4	−35.7	22.0
9	23	1m5fC_1m5hpmC	1m5hmC	−330.9	−338.2	−320.6	−329.2
	24	—	1m5caC	42.2	−9.0	20.4	−29.6
	25	1m5fC_1m5hpmC	1m5caC + 1m5hmC	−288.7	−347.2	−300.2	−358.8
	26	1m5fC + 1m5hphmC	1m5fC_1m5hphmC	−24.0	38.0	−31.4	28.6
	27	1m5fC_1m5hphmC	1m5caC + 1m5dhmC	−296.4	−355.9	−309.1	−369.0
	28	1m5fC + 1m5pcaC	1m5fC_1m5pcaC	−15.0	45.5	−22.9	34.9
	29	1m5fC_1m5pcaC	1m5caC + 1m5caC	−338.7	−394.9	−347.7	−402.8
	30	1m5fC + HOOH	1m5fC_HOOH	−6.0	36.6	−16.5	26.2
	31	1m5fC_HOOH	1m5caC + HOH	−342.4	−378.6	−351.8	−388.6
Orange	Endergonic (thermodynamically limiting) reactions						
Yellow	Exergonic reactions dependent on (thermodynamically limiting) reactions						
Green	Exergonic reactions or cycles						

*Note:* $\Delta H^{\mathrm{sol}}$ values refer to the 1 atm standard state, whereas $\Delta G^{\mathrm{sol}}$ values refer to the 1 mol L^−1^ standard state. Calculations were performed at the SMD/DFT (SMD(H_2_O)/(U)B3LYP‐D3/6–31+G(d,p)) and SMD/CC (SMD(H_2_O)/DLPNO‐CCSD(T)/CBS//SMD(H_2_O)/(U)B3LYP‐D3/6–31+G(d,p)) levels. All values are given in kJ mol^−1^.

In the present system, initiation may occur through hydrogen abstraction from the methyl group of 1,5dmC. Possible initiators include the HOO• radical, ozone, or decomposition of hydroperoxide intermediates. These pathways generate the carbon‐centered radical 5r_1,5dmC at the 5‐methyl substituent of the cytosine ring. According to the calculated thermodynamic data in Table 2, its formation through direct reaction of the substrate with triplet oxygen, •OO• (^3^O_2_), is strongly endothermic and endergonic. This is because such a step requires hydrogen abstraction by the relatively stable triplet oxygen molecule. The calculated reaction enthalpy is in good agreement with literature data for a similar reaction in the toluene system, where a value of ΔH≈170 kJ mol^−1^ has been reported. The authors of reference [24] also noted that such a high endothermicity makes direct substrate oxidation by triplet oxygen an unlikely pathway for autoxidation initiation.

This example mainly illustrates how thermodynamically difficult it is to initiate the reaction with molecular oxygen. In real systems, radical chain processes may start through other pathways. In particular, air may contain trace amounts of ozone formed in photochemical processes. Ozone is a much more reactive oxidizing agent and may initiate radical formation upon reaction with organic molecules, thereby providing a possible entry point into chain oxidation reactions [45]. In addition, reactions of ozone with organic compounds can lead to the formation of hydroperoxide, peroxide, and radical intermediates that further participate in typical autoxidation chain mechanisms [46]. Therefore, when considering possible initiation pathways of autoxidation in systems exposed to air, the potential role of ozone as a more reactive oxidant should also be taken into account.

The formed carbon‐centered radical can further react with •OO• to produce a peroxyl radical; this step is exothermic (Table 2). However, because the number of particles decreases during O_2_ addition, the reaction free energy is less favorable than the corresponding reaction enthalpy. For reactions involving a change in the number of particles, the standard‐state correction ${\mathrm{\Delta G}}_{298K}^{1\mathrm{atm}\to 1M}$ does not cancel between reactants and products and therefore gives a nonzero contribution to the calculated reaction free energy.

In reaction 3, HAT leads to the formation of the hydroperoxide intermediate 1m5hpmC and regenerates the radical 5r_1,5dmC, thereby closing cycle 1. This step is slightly endothermic and endergonic (Table 2), because the C—H bond broken in the substrate is stronger than the O—H bond formed in the hydroperoxide product. As a result, regeneration of the carbon‐centered radical 5r_1,5dmC in this step is thermodynamically unfavorable. Nevertheless, this reaction is much less endothermic than the initiation step because one of the reactants is already a radical species. Therefore, if considered in isolation, the reverse reaction could dominate. However, within a chain mechanism, this step may still occur if the formed hydroperoxide is rapidly consumed in subsequent reactions, such as reaction 4. According to Coote and co‐workers, hydrogen abstraction by peroxyl radicals can be thermodynamically unfavorable in many autoxidation systems; therefore, such propagation steps may contribute less to chain oxidation than assumed in the classical autoxidation scheme [47].

In 1m5hpmC, several bonds can in principle undergo homolytic cleavage, with the calculated bond strengths following the trend BDE(O—O) < BDE(C—H) < BDE(OO—H) (Table 1). Thus, the O—O bond is the weakest bond and represents the most accessible cleavage pathway. For example, reaction 4 is exothermic and exergonic and involves reaction of 1m5hpmC with 5r_1,5dmC, accompanied by cleavage of the weak O−O bond and leading to the formation of more stable products, namely 1m5hmC and the alkoxyl radical 5hor_1m5hmC. The alkoxyl radical 5hor_1m5hmC can then abstract a hydrogen atom from another molecule of 1,5dmC in reaction 5, regenerating the carbon‐centered radical 5r_1,5dmC and closing cycle 2. This step forms the oxidation product 1m5hmC.

Thus, the first two propagation cycles lead to the formation of hydroperoxide intermediates, regeneration of the central carbon‐centered radical 5r_1,5dmC, and formation of the first experimentally observed oxidation product, 1m5hmC. The species generated in these cycles may undergo further radical transformations, which are discussed in the following section.

### 3.5 Competing Oxidation Pathways From 1m5hpmC to 1m5fC

Further stages of autoxidation are shown in Figure 9 and lead to the formation of more oxidized cytosine derivatives, primarily the formyl derivative 1m5fC. These transformations involve a sequence of radical reactions in which hydroperoxide derivatives and the corresponding radical intermediates play an important role.

Cycle 3 begins with the hydroperoxide derivative 1m5hpmC. In reaction 6, HO• abstracts the C‐bound hydrogen atom from 1m5hpmC, forming H_2_O and the carbon‐centered radical 5r_1m5hpmC. In reaction 7, this unstable radical configuration, represented by a covalently bound transition‐state structure, decomposes to form the formyl derivative 1m5fC and the hydroxyl radical HO•, thereby closing cycle 3. Thus, this pathway corresponds to further oxidation of the substituent at position 5 and leads to the formation of one of the main reaction products.

An alternative pathway is represented by cycle 4. In reaction 8, HO• abstracts a hydrogen atom from the hydroperoxide O−H group of 1m5hpmC, forming H_2_O and the peroxyl radical 5hoor_1m5hpmC. In reaction 9, HAT occurs. This step is slightly endergonic because the C—H bond energy in 1m5hpmC is slightly higher than the O—H bond energy in the formed hydroperoxide. Nevertheless, the cycle terminates with a strongly exothermic step, reaction 10, which forms the formyl derivative 1m5fC and the hydroxyl radical HO• (Table 2).

Cycles 3 and 4 can also be viewed as alternative consumption pathways of the hydroperoxide intermediate 1m5hpmC relative to reaction 4 in cycle 2. While reaction 4 involves cleavage of the relatively weak O—O bond and leads to the hydroxymethyl product 1m5hmC, reactions 6 and 8 correspond to cleavage of the stronger C—H bond and O—H bond in the hydroperoxy group, respectively, and ultimately lead to the formyl product 1m5fC. These pathways require more reactive radical species, such as HO•, to initiate hydrogen abstraction from 1m5hpmC, but they lead to a different oxidation outcome. Thus, reactions 6 and 8 may act as competing pathways that consume 1m5hpmC before it is converted to 1m5hmC and instead channel the reaction sequence directly toward 1m5fC. Such competition may contribute to the experimentally observed accumulation of the formyl derivative and to the relatively efficient formation of 1m5fC compared with 1m5hmC.

### 3.6 Oxidation of 1m5hmC to 1m5fC

The oxidation of the hydroxymethyl derivative 1m5hmC to the formyl derivative 1m5fC proceeds through two interconnected radical cycles shown in Figure 10. Cycle 5 consists of two propagation steps. In reaction 11, the peroxyl radical 5hoor_1m5hphmC abstracts a hydrogen atom from 1m5hmC, yielding the hydroperoxide 1m5hphmC and the carbon‐centered radical 5r_1m5hmC. In reaction 12, 5r_1m5hmC adds •OO• to regenerate 5hoor_1m5hphmC, thereby closing cycle 5. The hydroperoxide 1m5hphmC can subsequently decompose to form the formyl derivative 1m5fC together with hydrogen peroxide, H_2_O_2_ (reaction 13), completing the first oxidation pathway from 1m5hmC to 1m5fC.

**FIGURE 10:**
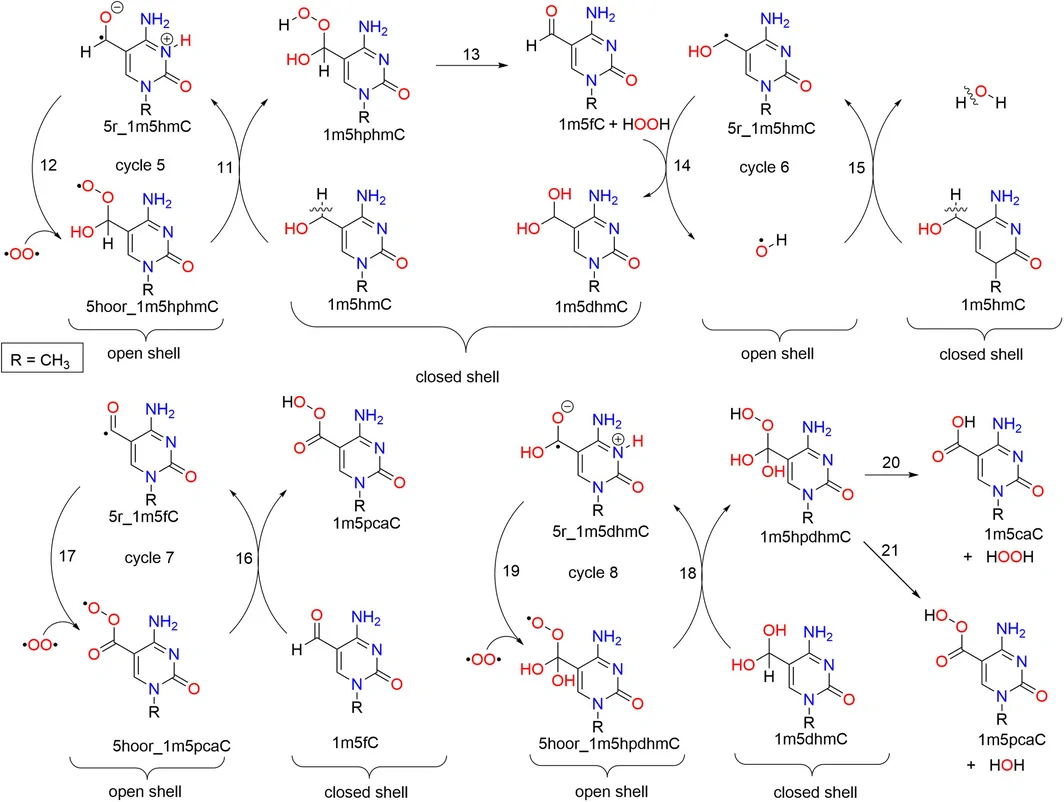
Chemically guided radical reaction network considered for the autoxidation of the substituent at position 5 in 1,5‐dimethylcytosine: Cycles 5–8. Stable closed‐shell products: 1m5fC and 1m5caC.

A second possible route for the oxidation of 1m5hmC to 1m5fC is represented by cycle 6, which illustrates how H_2_O_2_ formed in reaction 13 may be further consumed within the radical reaction network. In reaction 15, HO• abstracts a hydrogen atom from 1m5hmC, forming the carbon‐centered radical 5r_1m5hmC and H_2_O. The resulting carbon‐centered radical reacts with H_2_O_2_ in reaction 14 to form 1m5dhmC and regenerate HO•, thereby closing cycle 6. The resulting 1m5dhmC may subsequently dehydrate to 1m5fC and H_2_O.

### 3.7 Oxidation of 1m5fC to 1m5caC

Further oxidation of the formyl derivative 1m5fC leads to the formation of the carboxyl derivative 1m5caC. The corresponding stages of the reaction network are shown in Figure 10 and include two alternative radical cycles.

Cycle 7 consists of a hydrogen‐atom‐transfer step followed by O_2_ addition. In reaction 16, the peroxyl radical 5hoor_1m5pcaC abstracts the formyl hydrogen from 1m5fC, yielding the carbon‐centered radical 5r_1m5fC and the percarboxylic acid 1m5pcaC. In reaction 17, 5r_1m5fC adds •OO• to regenerate 5hoor_1m5pcaC, thereby closing cycle 7.

Cycle 8 involves further transformations of the hydrated, dihydroxymethyl form of the formyl derivative, 1m5dhmC. This pathway was included because hydration of 5‐formylcytosine derivatives has been shown experimentally and computationally to be possible, although the equilibrium concentration of the hydrate is low for unmodified 5fC derivatives [9, 20]. In addition, recent experimental and computational work on the oxidation of 5‐formyl nucleobases showed that hydrate formation can influence the subsequent oxidation pathway, especially when the hydrated form is thermodynamically accessible. In the present calculations, the hydrated form 1m5dhmC is also relevant because it has a substantially lower BDE(C—H) than the corresponding formyl derivative 1m5fC, largely due to tautomeric stabilization of the resulting 5r_1m5dhmC radical, making hydrogen abstraction from this species thermodynamically more favorable.

In reaction 18, the peroxyl radical 5hoor_1m5hpdhmC abstracts a hydrogen atom from 1m5dhmC, yielding 1m5hpdhmC and the carbon‐centered radical 5r_1m5dhmC. In reaction 19, this radical adds •OO• to regenerate 5hoor_1m5hpdhmC, thereby closing cycle 8. The following steps lead either to 1m5caC with the concomitant formation of hydrogen peroxide, H_2_O_2_ (reaction 20), or to the formation of the percarboxylic acid 1m5pcaC with the release of water (reaction 21).

According to the calculated thermodynamic data, cycle 8 is overall exergonic (Table 2), but it is thermodynamically limited by the positive free energy required for the formation of the hydrated form 1m5dhmC. Thus, this pathway should not be interpreted as the dominant oxidation route under all conditions, but rather as a thermodynamically plausible route that may become relevant when the hydrated form is accessible. In addition, analysis of the BDE(O—H) (Figure 7) and BDE(O—O) values (Figure 8) shows that among all considered hydroperoxide species the molecule 1m5pcaC has the strongest O—H and O—O bonds. This suggests that this percarboxylic acid may be comparatively less prone to homolytic O—H or O—O bond cleavage and could therefore persist longer than the other hydroperoxide intermediates considered.

### 3.8 Hydroperoxide Addition to the Formyl Group of 1m5fC and Formation of 1m5caC

The final stage of the considered reaction network is shown in Figure 11 and includes an additional radical cycle as well as several possible reactions between hydroperoxide intermediates and the formyl derivative 1m5fC.

**FIGURE 11:**
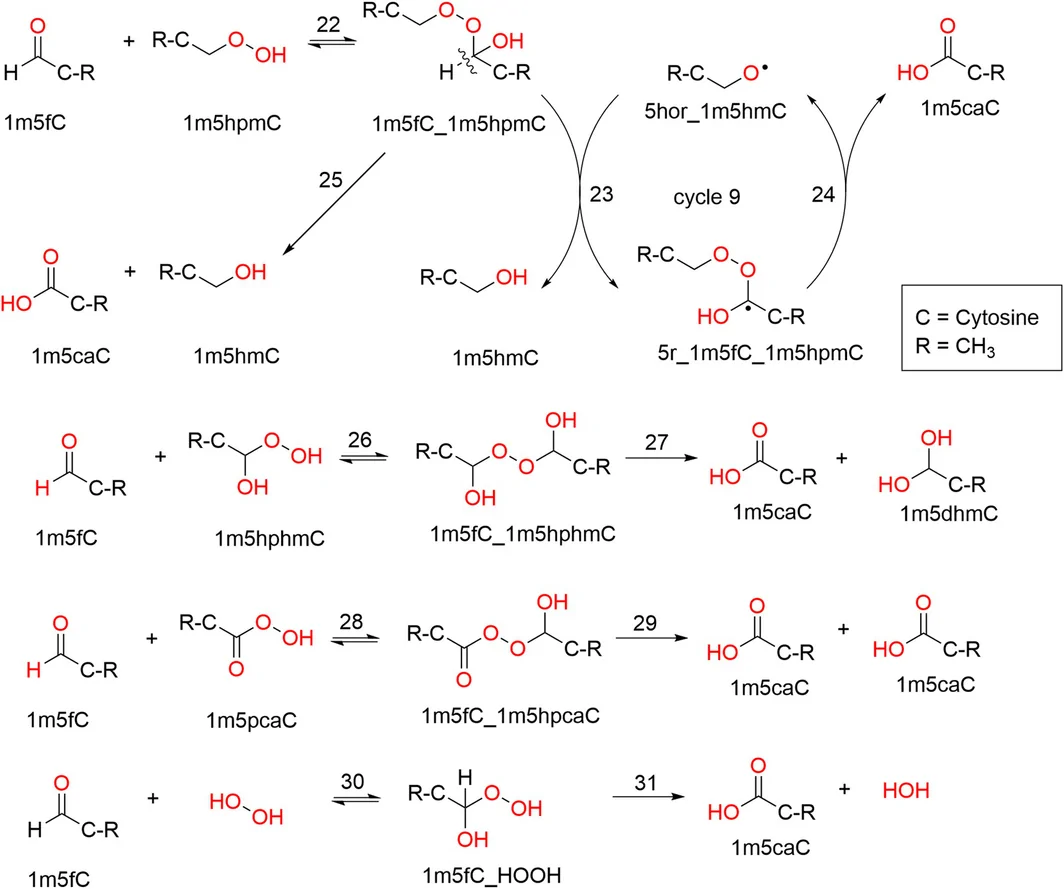
Chemically guided radical reaction network considered for the autoxidation of the substituent at position 5 in 1,5‐dimethylcytosine: cycle 9 and selected reactions between hydroperoxide intermediates and 1m5fC. Stable closed‐shell product: 1m5caC.

Cycle 9 begins with the formation of an adduct between 1m5fC and the hydroperoxide derivative 1m5hpmC (reaction 22). According to the calculated thermodynamic data, this cycle is overall exergonic (Table 2), but it is thermodynamically limited by the positive free energy required for the formation of the intermediate 1m5fC_1m5hpmC. Once formed, the adduct may either proceed through reactions 23–24 or decompose directly to 1m5caC and 1m5hmC in reaction 25; the latter process is strongly exergonic (Table 2).

The intermediate 1m5fC_1m5hpmC may undergo homolytic cleavage (reaction 23) to form the carbon‐centered radical and the hydroxymethyl derivative 1m5hmC. Further transformation of this radical leads to the formation of the carboxyl product 1m5caC (reaction 24), which completes this cycle.

In addition, several alternative reactions between 1m5fC and different hydroperoxide species were considered. In reaction 26, the formyl derivative reacts with the hydroperoxide 1m5hphmC to form the intermediate 1m5fC_1m5hphmC, which then decomposes to give 1m5caC and 1m5dhmC (reaction 27). In reaction 28, a similar interaction occurs between 1m5fC and the percarboxylic acid 1m5pcaC to form the adduct 1m5fC_1m5pcaC, whose decomposition produces two molecules of 1m5caC (reaction 29).

Finally, the reaction between 1m5fC and hydrogen peroxide, H_2_O_2_, was also considered (reaction 30). In this case, the intermediate 1m5fC_H_2_O_2_ is formed, which then decomposes to produce 1m5caC and a water molecule (reaction 31). It should be noted that reactions 26, 28, and 30 are endergonic, but their products further decompose exergonically to form 1m5caC (Table 2).

The calculations indicate that interactions between the formyl derivative and different hydroperoxide intermediates may provide several thermodynamically accessible routes toward the carboxyl product 1m5caC. Overall, the results suggest that selected pathways within the considered autoxidation network of 1,5dmC are thermodynamically feasible within the present model. However, conclusions regarding biological systems should be made with caution, since the native molecular environment of cytosine is not represented in the present model.

### 3.9 Transition‐State Calculations for Hydrogen Abstraction

Representative transition‐state calculations were carried out for H‐abstraction by CH_3_OO• and CH_3_O•, used as compact models of peroxyl and alkoxyl radicals, respectively, at the SMD/DFT level (Table 3).

**TABLE 3:** Boltzmann‐averaged activation enthalpies, $\Delta H^{‡\mathrm{sol}}$, and Gibbs free energies, $\Delta G^{‡\mathrm{sol}}$, for hydrogen abstraction from model methylated cytosine derivatives by CH_3_OO• and CH_3_O• radicals.

Reactant	Model radical	SMD/DFT		SMD/CC/TZ		Corresponding reaction
		$\Delta H^{‡\mathrm{sol}}$	$\Delta G^{‡\mathrm{sol}}$	$\Delta H^{‡\mathrm{sol}}$	$\Delta G^{‡\mathrm{sol}}$	
1,5dmC	CH_3_OO•	32.1	78.5	65.2	103.3	3
1m5hpmC		37.1	81.3	72.1	110.7	9
1m5hmC		25.4	70.8	59.8	100.5	11
1m5fC		36.4	79.7	65.1	100.3	16
1m5dhmC		27.0	72.2	73.5	112.6	18
1,5dmC	CH_3_O•	−4.5	37.1	26.3	61.8	5
1m5hpmC		1.1	39.6	29.9	63.8	—
1m5hmC		−2.8	38.6	19.1	58.4	15
1m5fC		−1.9	36.9	29.1	62.9	—
1m5dhmC		−5.2	35.9	35.9	73.9	—

*Note:* CH_3_OO• and CH_3_O• were used as small model radicals representing peroxyl and alkoxyl radicals, respectively. $\Delta H^{‡\mathrm{sol}}$ values refer to the 1 atm standard state, whereas $\Delta G^{‡\mathrm{sol}}$ values refer to the 1 mol L^−1^ standard state. All values are given in kJ mol^−1^ and were calculated at the SMD/DFT (SMD(H_2_O)/(U)B3LYP‐D3/6–31+G(d,p)) level, with additional SMD/CC/TZ (SMD(H_2_O)/DLPNO‐CCSD(T)/cc‐pVTZ//SMD(H_2_O)/(U)B3LYP‐D3/6–31+G(d,p)) single‐point energies.

For CH_3_OO•, the calculated $\Delta G^{‡\mathrm{sol}}$ values range from 70.8 to 81.3 kJ mol^−1^. For CH_3_O•, the corresponding barriers are substantially lower, 35.9–39.6 kJ mol^−1^. Additional SMD/CC/TZ (SMD(H_2_O)/DLPNO‐CCSD(T)/cc‐pVTZ//SMD(H_2_O)/(U)B3LYP‐D3/6–31+G(d,p)) single‐point calculations give higher absolute $\Delta G^{‡\mathrm{sol}}$ values, but preserve the qualitative result that H‐abstraction by CH3O• has lower barriers than H‐abstraction by CH3OO•. Thus, H‐abstraction by the alkoxyl radical is kinetically more accessible than by the peroxyl radical within the present model reactions. Within each radical class, however, the barriers vary only moderately across the investigated substrates. This suggests that the relative reactivity trends within the investigated model reactions may be influenced more strongly by the thermodynamics of radical formation and product‐radical stability than by large substrate‐specific differences in the calculated activation barriers.

A possible reason is that the stabilizing effects that are fully expressed in the product radicals are not yet fully developed in the transition state. In the H‐abstraction transition state, the transferred hydrogen atom is shared between donor and acceptor, so the TS does not fully reflect the additional stabilization of the final radical by the oxygen‐containing substituent. This interpretation is consistent with the Hammond postulate and Marcus‐type descriptions of HAT reactions: If the H‐transfer transition state is relatively early, it should be closer to the reactants than to the products, and differences in product‐radical stability should therefore be expressed more strongly in the reaction thermodynamics than in $\Delta G^{‡\mathrm{sol}}$ [48, 49, 50].

The present TS calculations are representative and do not constitute a full kinetic model of the complete radical network. Such a model would require transition states and reaction‐path calculations for all elementary steps and relevant radical conformers. Nevertheless, the results provide a useful kinetic check for the key H‐abstraction steps and support the thermodynamic interpretation developed above (Table 3).

## 4 Conclusions

The autoxidation pathways of the substituent at position 5 in 1,5‐dimethylcytosine were investigated as a chemically guided thermodynamic reaction network based on elementary steps known from radical autoxidation chemistry. The considered reaction network parallels classical hydrocarbon autoxidation, in particular toluene autoxidation, but also reflects the specific features of methylated cytosine derivatives, including heteroatom substitution, solvation effects, tautomerism, hydration of formyl derivatives, and protonation.

The calculated thermodynamic data show that direct initiation by triplet molecular oxygen, •OO• (^3^O_2_), is strongly endergonic and is therefore unlikely to represent an efficient initiation pathway. This result is consistent with previous thermochemical analyses of hydrocarbon autoxidation. In contrast, more reactive oxidants, such as ozone, may provide alternative initiation routes, as suggested by previous studies of autoxidation chemistry.

Bond dissociation energy analysis shows that hydrogen abstraction from the 5‐methyl substituent is the most thermodynamically accessible radical‐forming step among the considered abstraction sites in 1,5‐dimethylcytosine. The C—H bond strength depends strongly on the substituent structure and oxidation state. Formation of the 1m5dhmC‐derived radical is particularly favorable, whereas the formyl derivative 1m5fC has a stronger C—H bond and is less prone to direct hydrogen abstraction. Protonation systematically increases the calculated BDE(C—H) values and therefore reduces the thermodynamic tendency for carbon‐centered radical formation.

Analysis of O—H and O—O bond dissociation energies further shows that hydroperoxide intermediates can play an important role in the reaction network. The O—O bonds in hydroperoxy‐containing species are substantially weaker than the corresponding O—H bonds, making homolytic O—O cleavage a thermodynamically accessible decomposition pathway. Among the considered hydroperoxy‐containing species, the percarboxylic acid 1m5pcaC has the strongest O—H and O—O bonds and may therefore be comparatively less prone to homolytic decomposition.

Most reactions within the considered autoxidation network are exergonic; however, several groups of reactions are thermodynamically limiting. The first major limitation is radical initiation, especially direct hydrogen abstraction from 1,5dmC by triplet molecular oxygen, which is strongly endergonic and therefore unlikely to provide an efficient entry point into the chain process. A second bottleneck is associated with selected peroxyl‐radical HAT steps, including oxidation of 1,5dmC to 1m5hpmC and subsequent oxidation of 1m5hpmC to 1m5fC. In both cases, the peroxyl radical abstracts a hydrogen atom from a substrate C—H bond, giving slightly positive ΔG^sol^ values. Finally, nucleophilic addition of hydroperoxides or hydrogen peroxide to the formyl group of 1m5fC is generally endergonic, although subsequent decomposition of the resulting adducts can exergonically lead to the carboxyl product 1m5caC. Thus, the main thermodynamic bottlenecks of the considered reaction network are initiation, peroxyl‐radical HAT steps, and hydroperoxide addition to the formyl group.

The calculated reaction network provides a possible thermodynamic explanation for the experimental observation that the formyl derivative can accumulate during nonenzymatic oxidation of methylated cytosine derivatives. In a simple sequential oxidation picture, the hydroperoxide intermediate 1m5hpmC would first be converted to the hydroxymethyl product 1m5hmC, which would then undergo further oxidation to 1m5fC. However, the present calculations show that alternative radical pathways for the consumption of 1m5hpmC are thermodynamically feasible within the considered network, including pathways that bypass 1m5hmC formation and lead directly to the formyl product 1m5fC. In particular, reactions involving cleavage of the C—H bond or the O—H bond in the hydroperoxy group of 1m5hpmC provide competing routes toward 1m5fC rather than toward 1m5hmC. Such competition may reduce accumulation of the hydroxymethyl intermediate and channel the reaction sequence toward the formyl derivative, thereby partially rationalizing the experimentally observed accumulation of 1m5fC.

Representative transition‐state calculations for hydrogen abstraction by CH₃OO• and CH₃O• provide an additional kinetic check of the thermodynamic interpretation. The calculated activation free energies show that H‐abstraction by the alkoxyl model radical CH₃O• is substantially more accessible than abstraction by the peroxyl model radical CH₃OO•. However, within each radical class, the substrate‐dependent variation in ΔG^‡sol^ is moderate. This suggests that, within the investigated model reactions, the substrate‐dependent reactivity trends may be expressed more strongly in radical thermodynamics and bond dissociation energies than in the calculated H‐abstraction barriers.

Overall, the present results support the thermodynamic feasibility of selected nonenzymatic autoxidation pathways of the substituent at position 5 in 1,5‐dimethylcytosine, leading to the corresponding hydroxymethyl, formyl, and carboxyl oxidation products. The considered reaction network should not be interpreted as a complete kinetic model of all possible radical reactions in solution. Rather, it provides a thermodynamic framework for identifying plausible radical pathways, limiting steps, and chemically relevant intermediates. Additional radical reactions, explicit microsolvation effects, DNA‐environment effects, and enzymatic pathways are not excluded. Future work including extended transition‐state searches, explicit solvent models, and kinetic simulations will be required to establish quantitative reaction rates and product distributions.

## Funding

This work was supported by Deutsche Forschungsgemeinschaft (325871075).

## Data Availability

The data that support the findings of this study are openly available in TUHH TORE at https://doi.org/10.15480/882.17447.
